# Permethrin Resistance in *Aedes aegypti* Affects Aspects of Vectorial Capacity

**DOI:** 10.3390/insects12010071

**Published:** 2021-01-14

**Authors:** Tse-Yu Chen, Chelsea T. Smartt, Dongyoung Shin

**Affiliations:** Florida Medical Entomology Laboratory, Department of Entomology and Nematology, University of Florida, Vero Beach, FL 32962, USA; papilioninae@ufl.edu

**Keywords:** permethrin resistance, *Aedes aegypti*, vector competence, *kdr*, survival time, vectorial capacity

## Abstract

**Simple Summary:**

Pyrethroids have been applied as a major type of insecticide targeted at mosquitoes, many of which are key vectors in the transmission of several flaviviruses. Resistance to pyrethroids has emerged and become a worldwide threat to mosquito control. Pyrethroid resistance is usually accompanied by knockdown resistance (*kdr*) where the voltage gated sodium channel gene is mutated. We selected a permethrin resistant (p-s) *Aedes aegypti* population from Florida and, along with its parental population, examined two mutation sites, V1016I and F1534C. The data showed the p-s population had the most homozygous mutations which correlated to the permethrin resistance level. To assess the risk of disease transmission, two parameters affecting vectorial capacity were checked. The p-s population showed the longer survival time and higher dissemination rate for dengue compared to its parental population. The results showed that the potential threat of vector-borne diseases was increased in areas with resistant mosquitoes.

**Abstract:**

*Aedes aegypti*, as one of the vectors transmitting several arboviruses, is the main target in mosquito control programs. Permethrin is used to control mosquitoes and *Aedes aegypti* get exposed due to its overuse and are now resistant. The increasing percentage of permethrin resistant *Aedes aegypti* has become an important issue around the world and the potential influence on vectorial capacity needs to be studied. Here we selected a permethrin resistant (p-s) *Aedes aegypti* population from a wild Florida population and confirmed the resistance ratio to its parental population. We used allele-specific PCR genotyping of the V1016I and F1534C sites in the sodium channel gene to map mutations responsible for the resistance. Two important factors, survival rate and vector competence, that impact vectorial capacity were checked. Results indicated the p-s population had 20 times more resistance to permethrin based on LD50 compared to the parental population. In the genotyping study, the p-s population had more homozygous mutations in both mutant sites of the sodium channel gene. The p-s adults survived longer and had a higher dissemination rate for dengue virus than the parental population. These results suggest that highly permethrin resistant *Aedes aegypti* populations might affect the vectorial capacity, moreover, resistance increased the survival time and vector competence, which should be of concern in areas where permethrin is applied.

## 1. Introduction

Mosquitoes are the most common vectors of parasitic and viral diseases. Mosquito-borne diseases have a tremendous human impact, resulting in over one million deaths every year. The *Aedes aegypti* mosquito is the primary vector of many arboviruses, including yellow fever, dengue (DENV), chikungunya, and Zika viruses [1,2]. The application of insecticides is a widely used approach to control mosquitoes; however, insecticide use leads to the development of insecticide resistance within mosquito populations and also has detrimental effects especially on bees and aquatic organisms, in particular fish and crustaceans [3,4]. Therefore, there continues to be a need to understand the mechanisms that are involved in the development of insecticide resistance. This would enable use of traditional control methods while new non-chemical control strategies are being developed.

Mechanisms of insecticide resistance in mosquitoes include behavioral changes [5], alterations of the cuticle to reduce insecticide penetration [6], increased detoxification metabolism [7,8], and changes to target sites on sodium channel proteins [9]. Pyrethroids and DDT target the voltage-gated sodium channel of insect neurons. Single amino acid substitutions in the sodium channel have been associated with resistance to those insecticides [10]. This form of resistance, known as knockdown resistance (*kdr*), has been observed in several insect species including *Ae. aegypti* [11]. In many insect species, mutations related to both pyrethroid and DDT resistances have mainly been located in the IIS6 region of the voltage-gated sodium channel gene. The replacement of a leucine for a phenylalanine at amino acid 1014 (Leu1014Phe/Ser) was the most common [12,13]. In *Ae. aegypti*, a total of 12 point mutations have been detected [14,15] and 5 of them have been functionally examined including V410L, S989P, I1011M/V, V1016G/I, and F1534C [16,17]. Among those mutations, 1016 (Val to Ile or Gly) and 1534 (Phe to Cys) in the IIS6 and IIIS6 segments of the voltage-sensitive sodium channel gene, respectively [11,18,19,20], are widely distributed in Florida [21] and an issue for mosquito control.

The detoxification of insecticides in mosquitoes involves three major metabolic detoxification gene families: cytochrome P450s (P450s), esterases, and glutathione S-transferases (GSTs). P450s are one of the largest gene families in all living organisms and are known to respond to pyrethroid insecticide; esterase is more active against organophosphate [14]. GSTs are soluble dimeric proteins that are involved in the metabolism, detoxification, and excretion of a large number of endogenous and exogenous compounds [22,23]. The alteration of gene expression of insect P450s and GSTs results in increased enzymatic activities. Consequently, this alteration enhances the metabolic detoxification of insecticides and the development of insecticide resistance [7]. The increased gene expression levels in P450s have also been reported to be involved in the development of insecticide resistance [24].

Insecticide resistance is energetically costly and results in changes to the physiology and behavior of mosquitoes, which in turn may alter the vector competence. Many reports showed that changes in vector competence correlate positively or negatively with changes in pathogen transmission and other biological factors affecting vectorial capacity [25,26,27]. Vectorial capacity refers to the potential of a vector to transmit a pathogen. Development, life span, and fecundity are additional biological factors with strong impacts on vectorial capacity in *Ae. aegypti* and these factors are altered in insecticide-resistant populations [28,29]. Although chemical control remains the best method for controlling mosquitoes and the pathogens they transmit, insecticide-resistant mosquitoes can increase the risk of mosquito-borne disease transmission. This would greatly hinder mosquito control in its ability to effectively decrease the number of mosquito pests.

Here we selected a permethrin resistant *Ae. aegypti* population from field collected mosquitoes and tested both the survival time and vector competence which are parameters involved in vectorial capacity. The impact of insecticide resistance on mosquito biology—especially relating to mechanisms of vectorial capacity—can inform programs that operate to mitigate the impact of re-emerging mosquito-borne pathogens and the increase in insecticide resistance.

## 2. Methods

### 2.1. Mosquitoes

The *Aedes aegypti* larvae were collected in Key West, Florida in 2015 (Key West population). Field-collected mosquitoes were sorted by species and maintained in the laboratory under standard insectary conditions (28 °C, 14 h/10 h light/dark period, 70% relative humidity). Mosquito colonies are maintained by feeding female mosquitoes on blood from chickens following approved standard protocols (IACUC protocol 201807682) and eggs are collected [30]. The F_13_ to F_20_ generations from both populations were used in this study.

### 2.2. Selection for Permethrin Resistance in Ae. aegypti and Mortality Assessment

The adult Key West *Ae. aegypti* mosquitoes (4–6 days old), separated by sex as pupae to prevent mating as adults, were placed in bottles, and males and females exposed to permethrin using a CDC bottle bioassay [31,32,33]. The survivors were combined after the assay, allowed to mate and blood-fed to generate the population (p-s population) and maintained at 28 °C, 14 h/10 h light/dark period, 70% relative humidity. Thus, we exposed successive generations of mosquitoes to increasing concentrations of permethrin so that each generation suffered ~30% mortality from the exposure.

In the mortality experiment, 50 virgin females and males (4 to 6 days old) were placed separately in 1000 mL bottles coated with equivalent amounts of permethrin (43 μg) and the mortality counted after 90 min [32].

In the median lethal dose (LD50) study, p-s and Key West populations were used. Around 20 4- to 6-day-old virgin female mosquitoes were exposed to different concentrations of permethrin in coated 250 mL bottles. The mosquito knockdown percentage was recorded after 10 min of exposure and the permethrin concentration adjusted based on the population differences. The regression line was calculated to determine the LD50 for a single mosquito in each population. The resistance ratio was calculated by dividing the LD50 of the selected p-s population by the LD50 of susceptible parental Key West population.

### 2.3. Assessment of Phenotypic Insecticide Resistance in Selected Ae. aegypti

For genomic DNA extraction, single mosquito bodies from the p-s population and Key West populations were collected in 1.5 mL tubes containing 100 μL of 25 mM NaCl, 10 mM Tris-Cl pH 8.0, 1 mM EDTA, 200 μg/mL proteinase K, and homogenized using a pestle. After incubating the mixture for 30 min at 37 °C and inactivation of proteins with proteinase K at 95 °C for 5 min, the mixture was centrifuged at 12,000× *g* for 5 min, and the supernatant containing DNA used for PCR. The sodium channel gene amplification by PCR was used to detect V1016I, and F1534C mutations in each of the *Ae. aegypti* populations (Table S1).

For PCR analysis of *kdr* mutation sites, two reactions were assembled the same except that one contained a susceptible-specific primer and the other contained a mutant-specific primer [34] (Table S1). A PCR diagnostic test was performed with a total reaction volume of 25 μL, consisting of 100–200 ng genomic DNA, 2.5 μL 10× buffer, 0.5 mM dNTP mix, and 2.5 U JumpStart AccuTaq LA DNA Polymerase Mix. PCR conditions were one cycle of 96 °C for 30 s, then 35 cycles of 94 °C for 15 s, 60 °C for 30 s, and 68 °C for 1 min, followed by one cycle of 68 °C for 5 min. PCR products were checked by electrophoresis on 1.5% agarose gels in TEB buffer. Bands were visualized by GelRed^®^ staining on a Bio-Rad Gel Doc™ EZ System and the size determined from the 100 bp DNA Ladder (Invitrogen, Waltham, MA). The size of the PCR products for the detection of *kdr* alleles was 348 bp (V1016I) and 284 bp (F1534C), whereas the size of the products used as allele-nonspecific outer primers was 592 bp (V1016I) and 517 bp (F1534C) [34]. The PCR products from the outer primers were sequenced and confirmed and the mutant sites shown to be included in the fragments. The chi-squared test was applied to calculate the *p*-value.

### 2.4. Comparison of the Survivorship and Development between Permethrin-Resistant and Parental Populations

Survivorship was compared between the p-s population and the parental population, Key West. Adult mosquitoes that emerged on the same day were collected and used in this experiment. Twenty-five adult females from each population were placed in separate 8 oz soup cups (WebstaurantStore, Lancaster, PA, USA, Cat #760SOUP8KFT) covered with mesh for a total of 10 replicates and provided 20% sucrose water. Cups were placed in standard insectary conditions (28 °C, 14 h/10 h light/dark period, 70% relative humidity). The mortality was recorded each day until no more mosquitoes survived. Cox proportional-hazards model was used to analyze each population’s survivorship.

The larval development period was also compared between the p-s population and parental population, Key West. To hatch the eggs simultaneously, a piece of egg paper was placed in a flask containing water and connected to a vacuum to remove the air. After 30 min, 25 first instar larvae were placed in a plastic cup (9.5 cm diameter and 4 cm tall) containing 150 mL water and only fed once with 0.15 g of larvae food (a mixture of 1:1 brewer’s yeast (MP Biomedicals, Solon, OH, USA) and albumin (Acros Organics, NJ, USA). Each population had 10 replicated cups and were placed in the incubator (14:10 light cycle and 26 °C). Both the instar and mortality status were recorded every day. Once pupae were observed, the pupae were removed from the cup to a 50 mL Falcon tube (Thermo Fisher, Waltham, MA, USA, Cat #431176), and the sex was recorded after emergence. Tukey’s multiple comparisons test was used to analyze each population’s larval development time.

### 2.5. Comparison of Gene Expression Levels in Detoxification Genes Using qRT-PCR

To test the detoxification gene expression level, the p-s population and the Key West population were used. Five-day-old female mosquitoes were used and total RNA from pools of five female mosquitoes per population per group was extracted using Trizol Reagent (Invitrogen, Carlsbad, CA, USA) to determine the targeted gene expression [35]. Each group was replicated three times. The primers specific to the genes of interest (Table S2) were designed to determine changes in detoxification gene expression by Bio-Rad CFX96™ Real-Time PCR and the iTaq™ Universal SYBR Green One-Step Kit (Bio-Rad, Hercules, CA, USA). The related gene expression level was normalized to the expression of the *Ae. aegypti* ribosomal protein S7 gene (GenBank Accession # AY380336) [30]. All the PCR products were sequenced to confirm their identities (Eurofins, Louisville, KY, USA). The gene expression in each group was compared by delta-delta Ct value analysis. Wilcoxon test (GraphPad Prism 9) was used to calculate *p*-values and only *p* < 0.05 was counted as significantly different.

### 2.6. Infection of Ae. aegypti

Dengue virus (DENV-1, GenBank accession no. EU482591) was incubated with Vero cells for 5 days in the 37 °C incubator with 5% CO_2_ before mixed with bovine blood. Five to six-day-old female mosquitoes (100 per each population) were fed defibrinated bovine blood (Hemostat, Dixon, CA, USA) contained DENV-1 Mosquitoes were allowed to feed on the blood with DENV for 60 min from an artificial membrane feeding system (Hemotek, Discovery workshops, Blackburn, UK). Fully engorged females were separated from unfed females and the engorged females maintained in the incubator at 28 °C with a 20% sucrose solution for the duration of the experiment. There were fewer blood fed mosquitoes in the p-s population resulting in a smaller sample size compared to the parental population. After 14 days of incubation, the mosquito bodies and legs were collected separately as paired samples to check the infection rate and dissemination rate. RNA was extracted from individual bodies and legs by Trizol Reagent (Invitrogen, Carlsbad, CA, USA) and DENV detected using the iTaq™ Universal SYBR Green One-Step Kit (Bio-Rad, Hercules, CA, USA) with DENV specific primers on the Bio-Rad CFX96™ Real-Time PCR Detection System (Bio-Rad, Hercules, CA, USA) (Table S2) and following a standard protocol [35,36]. PCR products were sequenced to confirm their identity (Eurofins, Lexington, KY, USA) Fisher’s Exact Test was used to calculate the statistical significance.

## 3. Results

### 3.1. Mortality and LD50 for Permethrin between Permethrin-Resistant and Parental Populations

The p-s population was selected for permethrin resistance for 12 generations and generations F_13_ to F_20_ were used in the experiments. The resistance level to permethrin was assessed and compared between the p-s population and its parental population, Key West, using the standard CDC bottle bioassay. Mortality for the p-s population was six-fold lower than the Key West population at the new CDC diagnostic dose (43 µg/bottle) (Table 1) [32].

Permethrin median lethal dose (LD50) was tested for the Key West and p-s populations. The Key West population LD50 per mosquito was 16.88 ± 0.59 μg and 400.52 ± 69.64 μg for the p-s population. The p-s population was twenty times more resistant to permethrin than the Key West population (Table 2) and has a resistance ratio of 23. 7, as compared to the Key West population.

### 3.2. Assessment of the kdr Mutations in the Insecticide Resistant Ae. aegypti Population

Two mutations were identified from the genomic DNA from a subset of female and male *Ae. aegypti* of each population: V1016I (GTA-ATA) and F1534C (TTC-TGC) (Table 3). Both allele-nonspecific outer primer products for V1016I and F1534C were sequenced from both populations to confirm the mutation. The allele-specific PCR detected the mutation in both mutant sites, V1016I and F1534C. The F1534C loci contained the mutation in both the p-s population and Key West population (100%), however at the V1016I loci, the p-s population was 97% mutated, while only 64% in the Key West population. The difference at F1534C and V1016I between the two populations was significant (*p* < 0.0001, df = 2). Moreover, the population from Key West showed the most individuals as heterozygous (94%), and the p-s population showed mostly homozygous mutations (64%) for the mutation sites tested. The frequency of the resistance allele (R allele) of V1016I and F1534C in the p-s population was 0.86 and 0.76, respectively, and 0.32 and 0.55, respectively, in the Key West population.

### 3.3. Analysis of Survivorship and Larval Development in Populations of Ae. aegypti

The adult survivorship of the selected population (p-s) and parental population, Key West, was assessed to determine the effect of permethrin resistance on survivorship, a major component of vectorial capacity. Adult female mosquitoes from each population (250) were assessed and measurement of the average daily survival time revealed that the p-s population lived significantly longer than the parental population, Key West (Table 4).

In the larval development study using the same populations, however, both male and female development times were not significantly different between the populations (Table S3).

### 3.4. Comparison of Gene Expression Levels for Detoxification Genes in Both Ae. aegypti Populations

Several cytochrome P450 monooxygenases (P450) and glutathione S-transferase (GST) genes, which are detoxification genes for pyrethroids, were selected [18,37] (Table S2) and their gene expression assessed among the mosquito populations used in this study.

**Figure 1:**
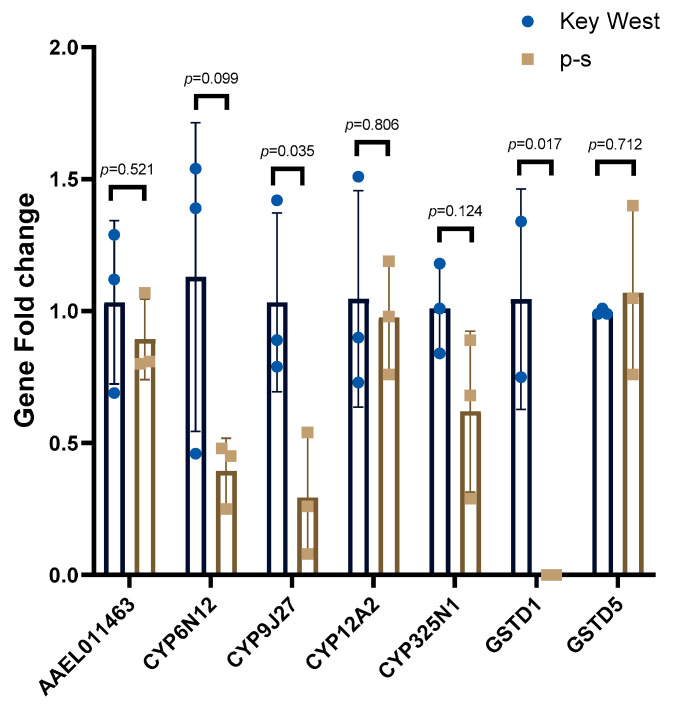
Relative detoxification gene expression between the p-s population and the Key West population. Three replicates of five pooled female mosquitoes were compared between p-s and Key West populations. The bar indicates the mean with standard deviation and the dots represent the expression level in each sample.

The Key West population was used to assess base-line detoxification gene expression levels and expression compared with the p-s population. One of the GST genes, *GSTD1* (AAEL001061), was significantly downregulated in p-s population (*p*-value = 0.0177, t = 4.753, df = 4). Another GST gene *GSTD5* (AAEL001071) was not expressed differently between the populations (*p*-value = 0.7123, t = 0.3961, df = 4) (Figure 1). Most of the P450 genes tested had no expression level differences in the p-s population compared to the Key West population but only *CYP9J27* (AAEL014616) was significantly downregulated (*p*-value = 0.0354, t = 3.124, df = 4) (Figure 1).

### 3.5. Vector Competence with DENV-1 Infection in Both Ae. aegypti Populations

Both p-s and Key West populations were fed with 6.94 logPFUE/mL of DENV-1. After 14 days, mosquitoes were dissected, bodies (infection rate) and legs (dissemination rate) were collected to detect DENV-1. Both populations had a high infection rate, where the p-s population was 100% and the Key West population was 96.8%. The p-s population had a significantly higher dissemination rate (71%) compared to the Key West population (30.7%) (Table 5).

## 4. Discussion

Resistance to permethrin in *Aedes aegypti* has been a worldwide issue for mosquito control [3,38]. In this study, we generated a laboratory selected permethrin- resistant *Ae. aegypti* population and checked the resistance level, evaluated survival and vector competence which influence vectorial capacity, a measure used to assess potential vector-borne diseases transmission risk, to evaluate influence of insecticide resistance. Here, we provide the first evidence of the impact of insecticide resistance in *Ae. aegypti* on the transmission of DENV.

The p-s population had higher resistance compared to the parental Key West population (Table 1) indicating the selection was successful. The permethrin LD50 study verified that the p-s population had significantly higher dose tolerance for permethrin (Table 2) and corroborates that this population is resistant.

It was previously shown that *Ae. aegypti* in Florida have different levels of resistance to permethrin and *kdr* mutations, and substitutions at positions 1016 and 1534 in the voltage-gated sodium channel gene were the most common [21]. Here we examined both *kdr* mutant sites in the permethrin selected *Ae. aegypti* population and the parental population and found the p-s population had a higher frequency of the resistant allele in both voltage-gated sodium channel gene sites, V1016I and F1534C (Table 3). The presence of an F1534C mutation is known to reduce the channel sensitivity to permethrin [39] and the combination of V1016I and F1534C alleles have more resistance to the pyrethroid deltamethrin than only the V1016I mutation [40]. Furthermore, the combination of V1016I and F1534C enhanced the resistance to both Type I and Type II pyrethroids [41]. Several field studies also indicated the existence of both V1016I and F1534C associated with pyrethroid resistance in mosquito populations around the world [21,42,43,44]. Besides the frequency of mutation, the p-s population had more homozygous mutations at both mutant sites than the Key West population (Table 3). Previous studies showed presence of the homozygous mutations of V1016I and F1534C resulted in higher resistance to deltamethrin than heterozygous mutants [40].

The elevated insecticide resistance affected adult longevity of the p-s population as these mosquitoes had a longer life span than the parental Key West population (Table 4). These findings are contrary to what was shown in previous studies where pyrethroid resistance in mosquitoes was shown to have negative effects on life-history traits including decreased adult longevity [29,45]. However, the previous study focused on field mosquitoes where our study utilized a lab selected permethrin resistant strain [29]. Additionally, longevity is one of the important factors in the measurement of vectorial capacity [46] because it allows infected mosquitoes to transmit the pathogenic virus for a longer period. The oxidative stress induced by mitochondrial ROS production influenced the adult survival of *Anopheles gambiae* [47], furthermore, the P450s activity was associated with oxidative stress stimulated by DDT and pyrethroids in *An. arabiensis* and *An. Funestus* [48]. Although the oxidative stress from P450s is essential to pyrethroid-resistance [49], some of the increased detoxification metabolism is initiated by insecticide contact rather than the result of an inherently high metabolism [18]. In this p-s population, perhaps the detoxification gene proteins might not be induced without permethrin exposure, decreasing the oxidative stress to maintain functions involved in longevity (Figure 1). However, further experiments with the p-s population will be needed to support this conclusion.

Insecticide resistance can be conferred by increased detoxification metabolism [50,51]. Resistant mosquito populations have also been shown to have alterations in the expression of the genes encoding the detoxification proteins [52,53]. Here we checked the expression of several detoxification genes and compared the levels between p-s and Key West populations (Figure 1). Both *CYP9J27* and *GSTD1* had significantly lower expression levels in the p-s population than the Key West population and could contribute to the adult longevity. This supports the previous conclusion that these genes might not be induced without permethrin exposure.

In the larvae development time experiment, both the male development time and the female development time were not significantly different across different populations (Table S3). Resistance to permethrin was shown to not impact pupation in select isofemale lines from a DDT- and permethrin-resistant *Ae. aegypti* population originally collected in Thailand [54] and supports finding from this study.

Another important parameter that may influence vectorial capacity is vector competence. Here we infected both p-s and Key West populations with DENV-1 to check the infection rate and dissemination rate (Table 5). Although both populations had a high infection rate, the virus must escape from the midgut barrier to be transmitted. The p-s population had a significantly higher dissemination rate than the parental population, Key West, and suggests that this population could be a better vector for DENV-1. However, DENV transmission was not compared between these populations and these conclusions are speculative. Although there were several studies that found the infection with the malaria parasite increased the probability of parasite transmission through increased sporozoite prevalence in insecticide resistant mosquitoes [55,56] and insecticide-resistant *Cx. quinquefasciatus* had an increase in transmission efficiency for West Nile virus [57], there have been few studies indicating enhancement of arbovirus transmission in insecticide resistant *Ae. aegypti.* Further work is ongoing to identify the potential mechanisms responsible for increased susceptibility to virus in the insecticide resistant population generated in this study. One potential reason for higher susceptibility to virus in the p-s population could be the low expression of immune-related pathways. Several immune pathways are known antiviral responses [58] and was downregulated in insecticide selected *Ae. aegypti* [59]. Suppression of the expression of immune response genes might be expected to be due to efforts needed to maintain the resistance phenotypes. This may result in resistant mosquitoes being more susceptible to outside pathogens. However, expression of immune response genes in this permethrin resistant population has not been investigated. The mosquito microbiota may also contribute to differences in susceptibility of the p-s population to virus as the different gut microbiota were shown present in mosquitoes that are susceptible and resistant to lambda-cyhalothrin [60] and it is known that the microbiome influences mosquito vector competence [61].

## 5. Conclusions

In conclusion, we selected the permethrin resistant *Ae. aegypti* from a Florida population and confirmed the resistance level from mortality and LD50 experiments. Homozygous *kdr* mutations at both nucleotides 1016 and 1534 of the voltage-gated sodium channel gene were characterized in the p-s population and might contribute to high resistance to permethrin. In fact, co-occurrence of multiple resistance mechanisms, such as the V1016G *kdr* mutation and overexpression of *CYP9J28*, was shown to be synergistic in conferring pyrethroid resistance in genetically engineered *Drosophila* [62]. We noticed the p-s population had a longer survival time in the adult stage, moreover, the dissemination rate of DENV-1 in the p-s population was significantly higher than its parental population, Key West. Both survival and vector competence are essential factors to describe the potential of a vector to transmit a pathogen. These results contribute crucial knowledge important to the mosquito control programs about the potential risk of emerging mosquito-borne diseases in areas where permethrin is regularly applied.

## Figures and Tables

**Table 1:** Mortality of *Ae. aegypti* populations following exposure to permethrin.

*Ae. aegypti* Population	No. Tested	% Mortality *
Key West	200	0.815 ± 0.087
p-s	420	0.235 ± 0.009

* Values are the mean ± SE.

**Table 2:** Toxicity of permethrin between the *Ae. aegypti* populations.

Population	No. Tested	LD50 ± SE (μg)
Key West	80	16.88 ± 0.59
p-s	45	400.52 ± 69.64

**Table 3:** Number of mutations in the sodium channel gene in populations of *Ae. aegypti*.

Population	No. Tested	*Kdr* Mutations	SS	RS	RR	Frequency of R Allele
p-s	39	V1016I	1	9	29	0.86
		F1534C	0	19	20	0.76
Key West	39	V1016I	14	25	0	0.32
		F1534C	0	35	4	0.55

**Table 4:** Analysis of survivorship of the *Ae. aegypti* populations.

Population	No. Tested	Average Survival Time
p-s	250	17.96 ^a^
Key West	250	11.80 ^b^

^a,b^ Levels not connected by the same letter are significantly different.

**Table 5:** Infection rate and dissemination rate of the *Ae. aegypti* populations after 14 days of DENV-1 infection.

	Infection Rate		Dissemination Rate	
Population	No. Tested	No. Body Infected (%)	No. Tested	No. Leg Infected (%)
p-s	38	38 (100%)	38	27 (71%) ^a^
Key West	95	92 (96.8%)	91	28 (30.7%) ^b^

^a,b^ Levels not connected by the same letter are significantly different.

## Data Availability

No new data were created or analyzed in this study. Data sharing is not applicable to this article.

## Supplementary Materials

The following are available online at https://www.mdpi.com/2075-4450/12/1/71/s1. Table S1, Primers used for the sodium channel gene mutation analysis. Table S2, Primer sequences for gene expression studies and detection of DENV. Table S3, The average pupation time for the *Ae. aegypti* populations.
