# Supplementary material for: Permethrin Resistance in Aedes aegypti Affects Aspects of Vectorial Capacity
Source: Insects. 2021 Jan 14;12(1):71. doi: 10.3390/insects12010071 (PMC7830706; doi:10.3390/insects12010071)
Supplement: Supplementary file 1 [file insects-12-00071-s001.pdf]

Table S1. Primers used for the sodium channel gene mutation analysis

Mutation	Primer	Sequence (5'→3')
V1016I	V1016G-For	GCCACCGTAGTGATAGGAAATC
	V1016G-Rev	CGGGTTAAGTTTCGTTTAGTAGC
	V1016G-V	GTTTCCCACTCGCACAGGT
	V1016G-I	GTTTCCCACTCGCACAGATA
F1534C	F1534C-For	GGAGAACTACACGTGGGAGAAC
	F1534C-Rev	CGCCACTGAAATTGAGAATAGC
	F1534C-F	GCGTGAAGAACGACCCGA
	F1534C-C	GCGTGAAGAACGACCCGC

Table S2. Primer sequences for gene expression studies and detection of DENV

Gene	Gene ID	Sequence (5'→3')
cytochrome p450 F	AAEL011463	TTTCGATGTACGGTTGGACA
cytochrome p450 R	AAEL011463	GCTTTCGATACGCTGGAGTC
CYP325N1 F	AAEL012770	GTACCTTGAAGCGCAAGAGG
CYP325N1 R	AAEL012770	TGTTCAGCATTCCTTGCTTG
CYP6N12 F	AAEL009124	TTCACTTGCGCGATCACTAC
CYP6N12 R	AAEL009124	TGCAGCAATTTCCTCAACAG
CYP9J27 F	AAEL014616	ACGGCAAGAAAATGATGGAC
CYP9J27 R	AAEL014616	CGGTTCCATGACTCTCCCTA
CYP12A2 F	AAEL002005	TACATCGTTGACTCCGGACA
CYP12A2 R	AAEL002005	CGAAGCGATCACTTTGTTGA

GSTD1 F	AAEL001061	GGTTCAGCTTGAGGAACTCG
GSTD1 R	AAEL001061	TGGCGTGGAGCTGAATCTTA
GSTD5 F	AAEL001071	GCAAGCCAAAGTCAACGAAAG
GSTD5 R	AAEL001071	CGTAGACCTGCTCGTGATAGTA
S7 F	AY380336	ACAAGAACCAGCAGACCAC
S7 R	AY380336	TCCGGGAATTCGAACGTAAC
DENV-1 F	EU482591	GACACCACACCCTTTGGACAA
DENV-1 R	EU482591	CACCTGGCTGTCACCTCCAT

Table S3. The average pupation time for the *Ae. aegypti* populations

	Total		males		females	
Population	Number	Average days	Number	Average days	Number	Average days
p-s	179	6.2521368	89	6.1916667	90	6.6956522
Key West	186	6.0243902	95	5.8581081	91	6.5263158
